# Identification of candidate genes of QTLs for seed weight in *Brassica napus* through comparative mapping among *Arabidopsis* and *Brassica* species

**DOI:** 10.1186/1471-2156-13-105

**Published:** 2012-12-06

**Authors:** Guangqin Cai, Qingyong Yang, Qian Yang, Zhenxing Zhao, Hao Chen, Jian Wu, Chuchuan Fan, Yongming Zhou

**Affiliations:** 1 National Key Laboratory of Crop Genetic Improvement, Huazhong Agricultural University, Wuhan, 430070, China; 2 Key Laboratory of Rapeseed Genetics and Breeding of Agriculture Ministry of China, Huazhong Agricultural University, Wuhan, 430070, China

**Keywords:** Brassicaceae, Rapeseed, *Arabidopsis*, Comparative mapping, QTL, Map-based cloning, Seed weight

## Abstract

**Background:**

Map-based cloning of quantitative trait loci (QTLs) in polyploidy crop species remains a challenge due to the complexity of their genome structures. QTLs for seed weight in *B. napus* have been identified, but information on candidate genes for identified QTLs of this important trait is still rare.

**Results:**

In this study, a whole genome genetic linkage map for *B. napus* was constructed using simple sequence repeat (SSR) markers that covered a genetic distance of 2,126.4 cM with an average distance of 5.36 cM between markers. A procedure was developed to establish colinearity of SSR loci on *B. napus* with its two progenitor diploid species *B. rapa* and *B. oleracea* through extensive bioinformatics analysis. With the aid of *B. rapa* and *B. oleracea* genome sequences, the 421 homologous colinear loci deduced from the SSR loci of *B. napus* were shown to correspond to 398 homologous loci in *Arabidopsis thaliana*. Through comparative mapping of *Arabidopsis* and the three *Brassica* species, 227 homologous genes for seed size/weight were mapped on the *B. napus* genetic map, establishing the genetic bases for the important agronomic trait in this amphidiploid species. Furthermore, 12 candidate genes underlying 8 QTLs for seed weight were identified, and a gene-specific marker for *BnAP2* was developed through molecular cloning using the seed weight/size gene distribution map in *B. napus*.

**Conclusions:**

Our study showed that it is feasible to identify candidate genes of QTLs using a SSR-based *B. napus* genetic map through comparative mapping among *Arabidopsis* and *B. napus* and its two progenitor species *B. rapa* and *B. oleracea*. Identification of candidate genes for seed weight in amphidiploid *B. napus* will accelerate the process of isolating the mapped QTLs for this important trait, and this approach may be useful for QTL identification of other traits of agronomic significance.

## Background

Rapeseed (*Brassica napus* L., AACC, 2n=38) is one of the world’s most important oil crops and provides not only edible oil for human diets, but also protein-rich feed for animals and raw materials for industrial processes such as biodiesel production. *B. napus* is an amphidiploid species derived from the hybridization of its two diploid progenitor species, *B. rapa* (AA, 2n=20) and *B. oleracea* (CC, 2n=18) [1]. Studies have shown that *Arabidopsis thaliana, B. napus*, *B. rapa* and *B. oleracea* have a common ancestor [2-6].

Seed weight is one of the three yield components (siliques per plant, seeds per silique and seed weight) of plant productivity of rapeseed and is also related to oil and protein content [7-9]. Extensive efforts have been made in mapping of the QTLs for seed size/weight in crop species [10-14], and genes governing seed size/weight have been cloned in model plants *Arabidopsis* and rice through mutant analysis and map-based cloning [15-18]. However, molecular cloning of seed size/weight genes in other crops, such as rapeseed, wheat and soybean lags behind due to the more complicated genome structures of these crops and limited availability of genome sequence information.

Quantitative genetic analysis in *B. napus* showed that seed weight has a relatively high heritability and may primarily be controlled by genes with additive effects [11,14,19-21]. Quijada *et al.*[22] detected three QTLs (located on N7, N17 and N19, respectively) for seed weight in different populations and environments, but no common QTL was identified. Udall *et al.*[23] found 6, 4 and 5 QTLs of seed weight in Hua DH, SYN DH and testcross populations, respectively, with only one QTL (located on N14) detected in all populations and environments. Shi *et al.*[21] mapped 159 QTLs of seed weight in TN DH and RC-F_2_ populations across 10 environments with only one major QTL (*qSW.A7-2*) identified in all environments. In our previous study [11], 9 QTLs for seed weight in a doubled haploid (DH) population of *B. napus* were identified, among which two major QTLs, *TSWA7a* and *TSWA7b*, were stably detected across years. Interestingly, seed weight QTLs on A7 were repeatedly detected in other studies with diverse genetic materials [11,14,21,24]. However, little is known about the candidate genes for those mapped QTLs, and so far only two genes, *BnMINI3a* and *BnTTG2a*, were assigned as the candidate genes for *TSWA5b* and *TSWA5c*[11]. It is thus crucial to develop procedures that can accelerate the process of mapped-based cloning by identification of candidate genes of those QTLs.

Comparative mapping among related species is a powerful tool for genetic studies by offering the possibility of transferring genomic information from well-studied species to more genetically complicated ones. This advantage is particularly obvious in Brassicaceae, because *Arabidpsis thaliana*, as a model plant for dicots, has completed genome sequence [25], and a wealth of functional genomics information. Much effort has been focused on the comparative analysis between *Brassica* species and *A. thaliana*. Several comparative maps in Brassicaceae have been constructed based on RFLP markers, cDNA clones from *Brassica* species [2,26-32]. Other types of markers, such as IP (intron polymorphism) markers from *Arabidopsis*[33], and gene specific markers based on *Arabidopsis* sequences were also used [34]. So far, no comparative mapping with *Arabidopsis* has been conducted with genetic maps based on SSR markers or other unknown sequence markers.

Wang *et al.*[32] constructed an integrated linkage map of *B. napus* using mainly SSR markers with the aid of other type of markers including RFLP, and then attempted to identify homologous loci in *Arabidopsis* to these SSR markers. However, <2% of the primer pairs had homology in *Arabidopsis*, of which only 50% agreed with those identified using the corresponding SSR clone sequences. In such a case, it was difficult for comparative studies within Brassicaceae only based on the SSR marker primer sequences [32]. On the other hand, in a comparative study based on 6, 5 and 6 BACs’ sequences from *B. napus*, *B. rapa*, and *B. oleracea*, respectively, it was found that most homologous loci in A and C genomes of *B. napus*, *B. rapa* and *B. oleracea* have colinear relationship with the same loci in *Arabidopsis*[35]. This finding implies that using the SSR markers homologous loci information in the *B. rapa* and *B. oleracea* genomes may bridge the comparative analysis of *B. napus* and *Arabidopsis*.

In order to transfer the gene information effectively from *Arabidopsis* to *B. napus*, we developed a procedure for comparative mapping among three *Brassica* species (*B. napus, B. rapa* and *B. oleracea*) and *Arabidopsis* based on a SSR linkage map in *B. napus*. By making use of the map, we identified the putative genes involved in seed weight/size regulation in *B. rapa* and *B. oleracea*, and mapped these genes onto the SSR-based *B. napus* genetic map. Such a seed weight/size gene distribution map will allow us to pinpoint candidate genes underlying seed weight QTLs, thus facilitating the genetic and molecular studies of seed weight control.

## Methods

### Plant materials and phenotypic evaluation

A DH population of 190 lines was produced from microspore culture with a F_1_ cross between SW Hickory (a spring-type *B. napus* variety) and JA177 (a winter-type *B. napus* pure line), and named the SJ-DH population. The population was used for genetic and QTL mapping. Seed weight of each plant from the population was measured based on 500 fully developed seeds with three replications. The average seed weight was converted to 1000-seed weight (TSW) for each individual plant. The means of TSW of 10–15 plants from each plot were used for trait evaluation of parents, F1 and SJ DH lines. The detailed information for the production of the population, field trials and sampling procedures for seed weight measurement has been described previously [11].

### Molecular marker, linkage map and QTL mapping

Primer sequences for SSR markers used for genetic mapping were described by Fan *et al.*[11] and the sequence information of newly added SSR markers is provided in Additional file 1: Table S1. Linkage analysis with all markers was performed using MAPMAKER 3.0 [36]. A minimum log likelihood of the odds (LOD) score of 11.0 and a maximum distance of 25 cM were used to group loci into linkage groups (LGs). Genetic distances between SSR loci were calculated using the Kosambi mapping function. The nomenclature of LGs follows the rules proposed by the Multinational *Brassica* Genome Project [37]. QTLs were detected using the composite interval mapping (CIM) procedure with the software QTL Cartographer V2.5 [38]. The parameters and methods for QTL mapping were described as Fan *et al.*[11].

### Identification of homologous colinear loci in *B. rapa* and *B. oleracea* genomes through e-PCR amplification with SSR primers from *B. napus*

To align the SSR loci on each of the *B. napus* LGs to the homologous loci in the *B. rapa* or *B. oleracea* genome, electronic PCR (e-PCR) [39] was performed with the primers of the SSR markers mapped on the *B. napus* LGs and the genomic sequences of *B. rapa* (version 1.1) [6,40] and *B. oleracea* (version 2011-06-30) [41] as templates. The parameters for e-PCR were set to allow three mismatches and one gap for a given primer pair. Amplicons produced from the e-PCR then were analyzed to determine their colinear relationship between the *B. napus* LGs and the chromosomes of *B. rapa/B. oleracea*.

An amplicon is regarded as a putative homologous colinear locus on the A- (*B. rapa*) or C- (*B. oleracea*) genome to the locus defined by the SSR marker used for the amplification on a particular *B. napus* LG. A homologous colinear locus was determined when only three or more amplicons on a same chromosome of *B. rapa/ B. oleracea* could be generated with the SSR primers from a single LG of *B. napus*. Such a criterion would allow to reduce the non-specific alignment among a LG in the genome of *B. rapa or B. oleracea,* and to determine the orientation of a linear fragment, which is required to establish the corresponding linear relationship between a *B. napus* LG and a *B. rapa/B. oleracea* chromosome*.*

To facilitate the process of identifying homologous colinear loci, a Perl script called e-PCRmap (Additional file 2) was written to analyze the results of e-PCR using the following formula:

$$M_{\mathrm{xy}}=\sum_{k=1}^{n}L_{\mathrm{yk}}$$

where *L* is the variable describing the status of e-PCR amplification, while *M* is the number of markers that fall onto the chromosomes of *B. rapa* and *B. oleracea* (with successful amplification), x is the LG of *B. napus*, and y is the chromosome of *B. rapa* and *B. oleracea,* k is the marker index of each LG*,* and n is the markers number of each LG. When a marker (k) on a LG (x) has one or more amplifications on a particular chromosome (y), *L*_yk_ is assigned 1, otherwise *L*_yk_ assigned 0. The formula calculates how many possible amplicons are produced with the primers for the SSR markers in a particular LG.

The script generates a list of possible homologous colinear loci on the chromosomes of *B. rapa* or *B. oleracea* for each linkage group of *B. napus*, the order of the homologous colinear loci were the same to SSR loci distribution on the LG. When a marker had multiple amplification loci on a same chromosome, the accurate position for a particular locus was determined manually by referring to the physical positions of its upstream and downstream amplicons.

### Mapping *Arabidopsis* homologous loci onto *B. napus* genome

The Perl script described above was used to extract the sequences of effective amplicons in the *B. rapa* and *B. oleracea* genomes. The amplicons’ sequences were used as queries in searching for *Arabidopsis* homologues using the BLASTn program [42] against TAIR10 [43] with an E-value of 1.0 as an initial identification of homologous loci in *Arabidopsis*. The less stringent E-value could allow more homologous loci included for the identification of conserved blocks.

The positions and gene loci of best-hits in *Arabidopsis* genome sequences database were collected and comparatively mapped onto the *B. napus* LGs. Identification of the conserved *Arabidopsis* genomic blocks [5] on the *B. napus* LGs was performed essentially as described by Parkin *et al.*[30]. A conserved block contained a minimum of three mapped SSR loci with at least two homologous loci from one of the 24 defined *Arabidopsis* bocks [5] every 10 cM in the *B. napus* genetic map.

### Identification of putative seed size/weight genes in *B. napus*

To identify and locate the putative seed size/weight genes in *B. napus*, the homologous sequences of seed size/weight genes in the databases of *B. rapa* and *B. oleracea*[40,41] were first searched with the BLASTn [42] program (E value <1E-20 when using *Arabidopsis* seed size genes as query, and E value <1E-10 with the genes from other crops as query). The resulting sequences from the search were firstly mapped onto the chromosomes of *B. rapa* or *B. oleracea* and then placed on the *B. napus* LGs based on the homologous colinear relationships between *B. napus* and *B. rapa/B. oleracea*.

### Gene cloning

Standard molecular cloning procedures [44] were followed to isolate the homologous genes of *Arabidopsis* in the parental lines of the SJ DH population. The genomic fragments corresponding to the *AtAP2* gene were amplified using the primer pairs of AP2F (5'-ATGTGGGATCTAAACGACTCACCA-3') and AP2R (5'-TCAAGAAGGTCTCATGAGAGAAGG-3'). The PCR products from the parental lines were sequenced by the BigDye Terminator Cycle Sequencing v3.1 (Applied Biosystems, Foster City, CA, USA), and the coding sequences were predicted with the software SEQUENCHER 4.1.2 (Gene Codes Corporation, Ann Arbor, MI, USA).

## Results

### Construction of the genetic linkage map and mapping of the QTLs for seed weight

**Figure 1:**
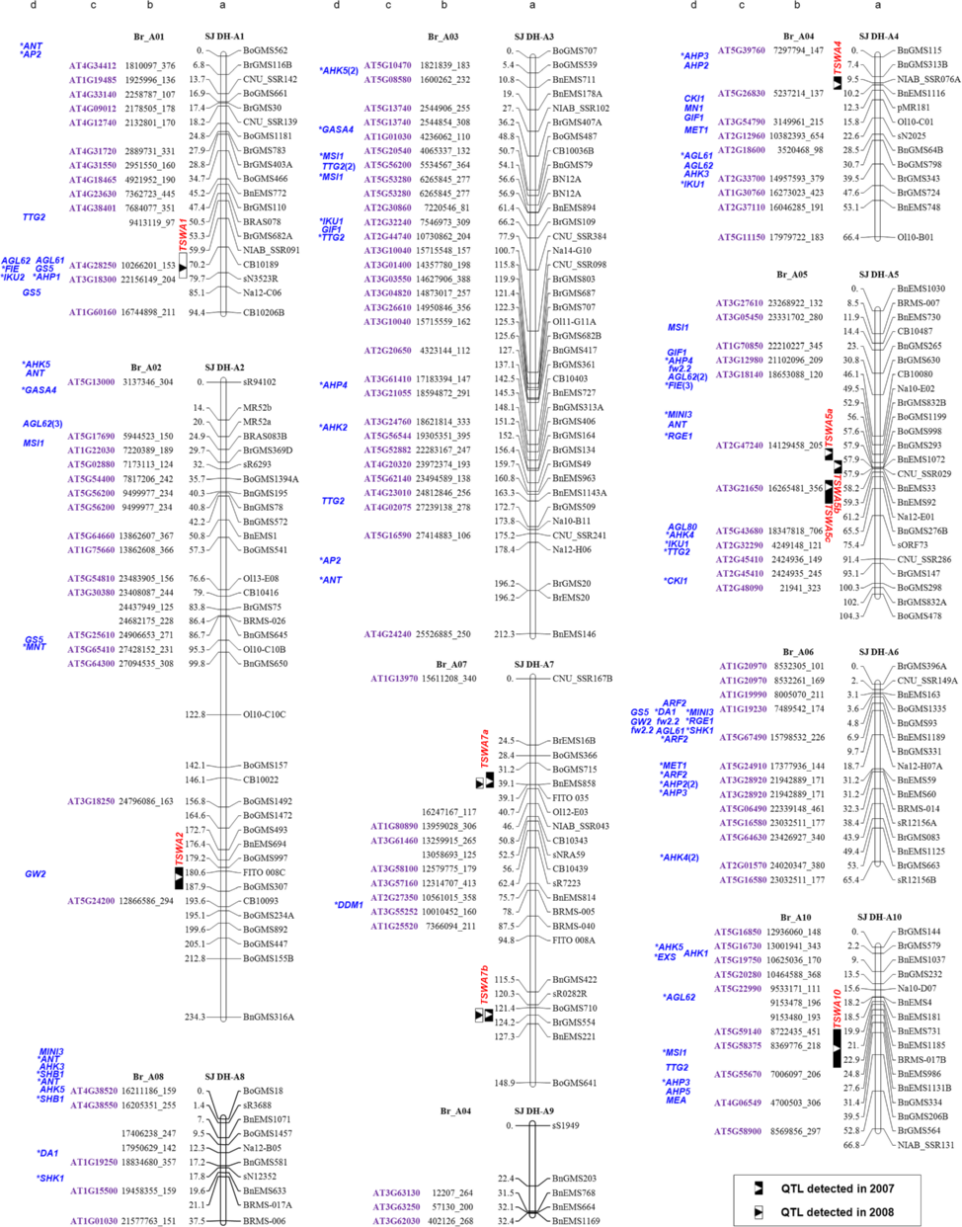
**Seed weight/size gene distribution map of *B. napus *(A genome).** Column a presents the genetic linkage groups of the SJ DH population. The nomenclature of LGs follows the rules proposed by the Multinational *Brassica* Genome Project [37]. Each of the LGs is represented with a vertical bar with the locus position (in cM) on the left and SSR loci names on the right. The QTLs information (peak, interval and name) of TSW were on the left-hand of the LGs. Column b lists the homologous colinear loci in *B. rapa* or *B. oleracea*. The numbers designate the physical position in *B. rapa* or *B. oleracea* chromosome with the length of amplification fragment. Column c is the *Arabidopsis* gene codes corresponding to the homologous loci. Column d lists the homologous genes of seed size or weight identified in *B. rapa* and *B. oleracea*. Genes that are in good fit into both *Arabidopsis* and *B. rapa*/*B. oleracea* physical positions are marked with asterisks. Numbers in brackets are the tandem repeat times of the tandem repeated genes.

**Figure 2:**
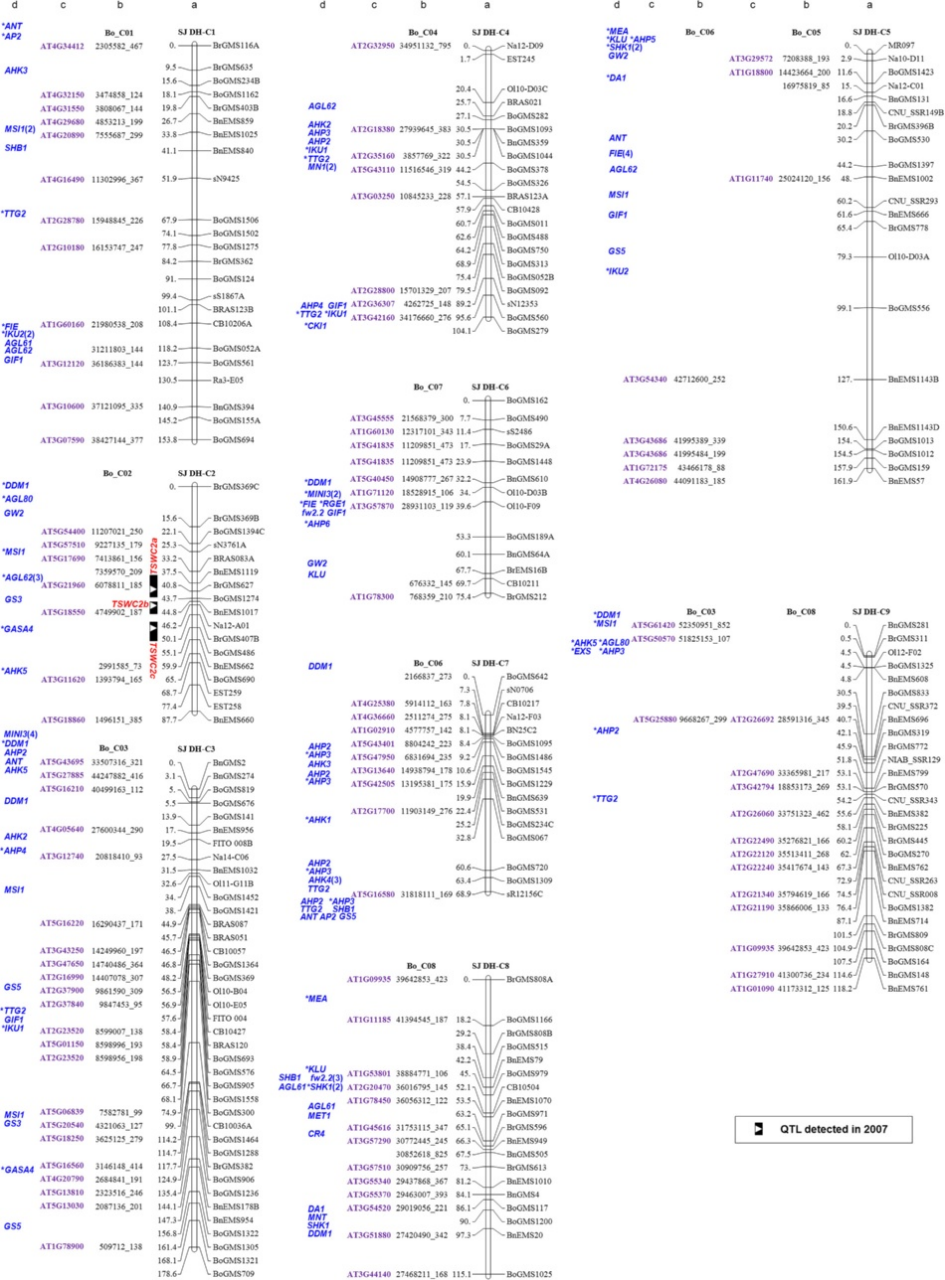
**Seed weight/size gene distribution map of *B. napus *(C genome).** Column a presents the genetic linkage groups of the SJ DH population. The nomenclature of LGs follows the rules proposed by the Multinational *Brassica* Genome Project [37]. Each of the LGs is represented with a vertical bar with the locus position (in cM) on the left and SSR loci names on the right. The QTLs information (peak, interval and name) of TSW were on the left-hand of the LGs. Column b lists the homologous colinear loci in *B. rapa* or *B. oleracea*. The numbers designate the physical position in *B. rapa* or *B. oleracea* chromosome with the length of amplification fragment. Column c is the *Arabidopsis* gene codes corresponding to the homologous loci. Column d lists the homologous genes of seed size or weight identified in *B. rapa* and *B. oleracea*. Genes that are in good fit into both *Arabidopsis* and *B. rapa*/*B. oleracea* physical positions are marked with asterisks. Numbers in brackets are the tandem repeat times of the tandem repeated genes.

Previously, we constructed a genetic map with 297 SSR markers for the SJ DH population [11]. In the present study, we expanded the SSR markers to 361 and re-constructed the map. In total, 397 SSR loci and 6 gene-specific markers were located to the new genetic map with 19 linkage groups (LGs). The markers covered a genetic distance of 2,126.4 cM (the previous map was 2,011.1 cM) with an average distance of 5.36 cM (the previous map was 6.15 cM) between markers (Figures 1, 2; Additional files 3, 4, 5 and 6: Figure S1-S4; Additional file 1: Table S1). The LGs corresponded to the 19 chromosomes of *B. napus* including A1-A10 (A genome) and C1-C9 (C genome) as determined by shared SSR markers in public genetic maps [37]. All SSR markers were evenly distributed across the whole genome of *B. napus* with 199 and 198 SSR loci on genome A and C, respectively (Table 1).

**Table 1:** **Number of e-PCR amplicons and corresponding homologous colinear loci in *B. rapa *(A genome) and *B. oleracea *(C genome) for *B. napus *linkage groups**

***B. napus***			***B. rapa***		***B. oleracea***	
**LG**	**SSR loci**	**Amplicons in *B. rapa *and *B. oleracea***	**Chr**	**Homologous colinear loci**	**Chr**	**Homologous colinear loci**
A1	19	35	A1	14	C1	13
A2	35	82	A2	18	C2	11
A3	39	71	A3	30	C3	15
					C6	7
A4	13	27	A4	9	C4	5
A5	24	69	A5	12	C5	4
			A6	6		
A6	16	73	A6	13	C5	4
					C6	7
A7	22	31	A7	10	C7	4
A8	10	23	A8	7	C3	3
					C8	3
A9	5	13	A4	3	C4	3
A10	16	40	A10	12	C9	9
**Subtotal**	**199**	**464**		**134**		**88**
C1	24	69	A1	10	C1	13
C2	17	46	A2	11	C2	9
C3	39	67	A3	18	C3	23
C4	21	75	A4	2	C4	8
C5	21	45	A1	3	C5	4
			A6	6	C6	5
C6	13	42	A7	3	C7	9
C7	16	39	A7	3	C6	10
C8	19	46	A9	11	C8	14
C9	28	52	A9	22	C3	3
					C8	12
**Subtotal**	**198**	**481**		**89**		**110**
**Total**	**397**	**945**		**223**		**198**

With the newly integrated map, the QTLs for TSW in the SJ DH population were re-scanned. A total of 12 QTLs of TSW were identified on 7 LGs (Additional file 7: Table S2), including three previously unidentified QTLs on LG C2 were detected in the year 2007, due to more molecular markers now available on the integrated map, which resulted in a higher density and better resolution in identification of subtle changes caused by genotypic effects. The distribution and effect of other QTLs, including two major QTLs (*TSWA7a* and *TSWA7b*) remained largely unchanged (Additional file 7: Table S2).

### Comparative mapping of *B. napus* and *Arabidopsis* mediated with *B. rapa* and *B. oleracea* genome sequences

With the primers (see Additional file 1: Table S1 for primer sequences) of the SSR markers mapped on the *B. napus* LGs, electronic-PCR (e-PCR) was performed using the genome sequence of *B. rapa* or *B. oleracea* as templates to obtain fragments amplified in respective genomes (amplicons). A computer program (e-PCRmap) was developed to operate the e-PCR process. In total, 945 amplified loci were obtained in the *B. rapa* and *B. oleracea* genomes with the primer sequences of 385 SSRs mapped on the SJ DH linkage map (Table 1 and Additional file 1: Table S1). From these analyses, 421 homologous colinear loci (amplicons that can be matched onto corresponding *B. napus* LGs) were identified (Table 1). Due to the highly colinearity between the A- and C- genome in *B. napus*, a SSR locus on a particular LG on *B. napus* may produce amplicons in both the *B. rapa* and *B. oleracea* genomes (Table 1; Additional files 3, 4, 5 and 6: Figure S1-S4).

Colinearity analysis between the *B. napus* LGs and the *B. rapa/B. oleracea* chromosomes showed the following three characteristics. First, some of *B. napus* LGs had high colinearity with the corresponding chromosomes of their progenitor species, *B. rapa* and *B. oleracea,* such as the LGs A1/C1, A2/C2, A3/C3, A4/C4 of *B. napus* that exhibited a sole colinear relationship with the chromosome A1, A2, A3, A4 in *B. rapa* and C1, C2, C3, C4 in *B. oleracea*, respectively (Figures 1 and 2; Additional file 3: Figure S1 and Additional file 4: Figure S2). Second, the LGs C6 and C7 of *B. napus* were colinear with the chromosome C7 and C6 of *B. oleracea*, respectively (Additional file 5: Figure S3). Such a corresponding relationship between LG C6 and chromosome C7 as well as LG C7 and chromosome C6 is likely resulted from the switched original labeling for *B. napus* linkage groups [45,46], as pointed out by Panjabi *et al.*[33]. Third, some of *B. napus* LG had a complex colinear relationship with the progenitor species due to the translocations during the evolution of the tetraploid species. Such a complexity is characterized by that a single *B. napus* LG may have syntenic segments from several chromosomes from *B. rapa* and /or *B. oleracea*. For example, the *B. rapa* chromosome A7 (BrA7) was found to have colinear segments on *B. napus* LGs C6 and C7, while the BrA6 segments existed in both LGs A6 and C5, and the BoC8 in LGs A8, C8 and C9, respectively (Figures 1 and 2; Additional file 5: Figure S3 and Additional file 6: Figure S4). In addition, LG C5 contained a homologous segment of BoC05 at its upper part and a homologous segment of BoC06 at its lower part (Figure 2). It was worth pointing out that *B. napus* LG C9 was largely colinear with BrA9, BoC8 and partly BoC3 simultaneously, rather than with BoC9. The LG A9 was short and thus no corresponding colinear segments could be matched with it. The short LG A9 is likely due to the low polymorphism between the two parental lines and thus fewer markers available for this linkage group. Such a result was consistent with our previous analysis [11].

By BLASTn analysis [42] against the *Arabidopsis* genome sequences (TAIR10) [43], the 421 homologous colinear loci from the *B. rapa* and *B. oleracea* genomes were aligned onto 398 homologous loci in *Arabidopsis*, with 212 loci from *B. rapa* and 186 from *B. oleracea*, respectively (Tables 1 and 2; Additional files 3, 4, 5 and 6: Figure S1-S4). These homologous loci were evenly distributed in the A and C genomes of *B. napus*, with 208 loci in LG A1-A10, and 190 loci in LG C1-C9 (Table 2). There were 23 amplicons without matched *Arabidopsis* homologous loci (Table 2 and Additional files 3, 4, 5 and 6: Figure S1-S4). In total, 71 *Arabidopsis* conserved blocks were resolved in the *B. napus* genome (Table 2 and Additional files 3, 4, 5 and 6: Figure S1-S4). The conserved blocks covered 1,411.3 cM of *B. napus* genetic linkage map, accounting for 66.4% of total length of the genetic map (Additional file 8: Table S3).

**Table 2:** **Distribution of conserved *Arabidopsis *genomic blocks on *B. napus *genetic map based on homology analysis between *B. napus *and *B. rapa/B. oleracea***

***B. napus***	**Chr**^**a**^	**Locus in *A. thaliana***						**ND**^**b**^	**Conservedblock**^**c**^
**LG**		**AtC1**	**AtC2**	**AtC3**	**AtC4**	**AtC5**	**Total**		
**A1**	A1	2		1	10		13	1	**2**
	C1	1		1	11		13		
**A2**	A2	2		2		12	16	2	**10**
	C2	2		2		7	11		
**A3**	A3	1	4	9	4	12	30		**6**
	C3	1	1	2	2	9	15		
	C6	1	1		3		5	2	
**A4**	A4	1	4	1		3	9		**3**
	C4	1	1	1		2	5		
**A5**	A5	1	5	5		1	12		**5**
	A6	5		1			6		
	C5	1		3			4		
**A6**	A6	4	1	2		6	13		**5**
	C5	2				2	4		
	C6		2	1		4	7		
**A7**	A7	3	1	4			8	2	**3**
	C7	2		1			3	1	
**A8**	A8	3			2		5	2	**3**
	C8	3					3		
	C3	2					2	1	
**A9**	A4			3			3		**1**
	C4			3			3		
**A10**	A10				1	9	10	2	**2**
	C9	1				7	8	1	
**Subtotal**		**39**	**20**	**42**	**33**	**74**	***B. rapa*: 125**	**14**	**40**
							***B. oleracea*: 83**		
**C1**	C1	1	2	3	6		12	1	**3**
	A1	1	1	4	4		10		
**C2**	C2			1		6	7	2	**2**
	A2	1	2			8	11		
**C3**	C3	1	5	3	2	12	23		**7**
	A3		6	4	1	7	18		
**C4**	C4		5	2		1	8		**3**
	A4				1	1	2		
**C5**	C5	2		1			3	1	**4**
	C6	1		3	1		5		
	A6	5					5	1	
	A1			3			3		
**C6**	C7	3		2		3	8	1	**2**
	A7	1		1	1		3		
**C7**	C6	1	1	1	2	4	9	1	**2**
	A7	1		1		1	3		
**C8**	C8	5	1	7			13	1	**3**
	A9	4	1	6			11		
**C9**	C8	3	8	1			12		**5**
	A9	7	9	5			21	1	
	C3					3	3		
**Subtotal**		**37**	**41**	**48**	**18**	**46**	***B. rapa*: 87**	**9**	**31**
							***B. oleracea*: 103**		
**Total**		**76**	**61**	**90**	**51**	**120**	**398**	**23**	**71**

^a^ Chromosome of *B. rapa* (A-genome) or *B. oleracea* (C-genome).

^b^ Not determined.

^**c**^ The number of unique blocks in a given *B. napus* LG. The conserved blocks were named according to Schranz *et al.*[5] and identified by the method described by Parkin *et al.*[30]. A conserved block contained a minimum of three mapped SSR loci with at least two homologous loci from one of the 24 defined *Arabidopsis* bocks [5] every 10 cM in *B. napus* genetic map.

Subsequently, a comparative map between *Arabidopsis* genome and the *B. napus* map based on SSR markers was constructed with the aid of *B. rapa/B. oleracea* genome sequences (Additional files 3, 4, 5 and 6: Figure S1-S4). In total, 385 SSR loci from *B. napus* exhibited synteny to *Arabidopsis* genes, but 114 SSR loci did not find any homologous regions in *Arabidopsis* (including 20 no amplification SSR loci). Altogether, 271 SSR loci on *B. napus* LGs were identified homologous to *Arabidopsis* (Additional files 3, 4, 5 and 6: Figure S1-S4; Additional file 1: Table S1).

### The comparative map can be used to identify candidate genes of mapped QTLs

**Figure 3:**
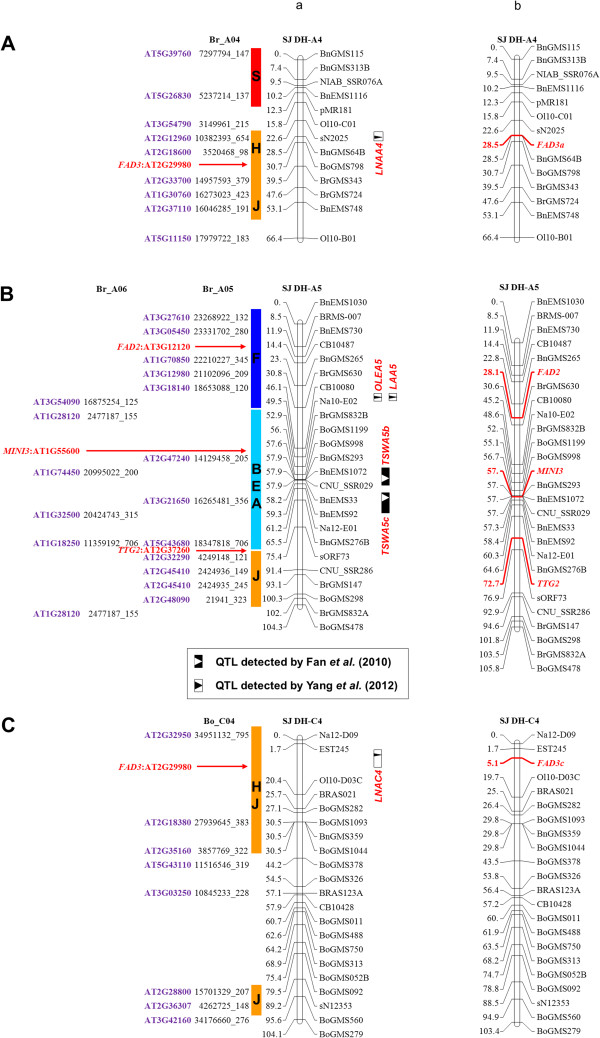
**Candidate genes underlying the QTLs identified through comparative mapping.** Candidate genes for mapped QTLs on LGs A4 (**A**), A5 (**B**) and C4 (**C**) are predicted with conserved *Arabidopsis* blocks mapped on *B. napus* map. Column a illustrates the prediction of the candidate genes. Previously mapped QTLs [11,47] are indicated on the right-hand side of the respective LG and the conserved *Arabidopsis* blocks (color bars with their names (letters) inside) on the left. The homologous loci in *B. rapa/B. oleracea* and *Arabidopsis* are listed next to the conserved blocks. Red arrows indicate the positions of the predicted candidate genes. Column b presents LGs A4 (**A**), A5 (**B**) and C4 (**C**) with newly added gene-specific markers.

Previously, we identified several QTLs for seed weight and fatty acid content with the SJ-DH population, and cloned the candidate genes underlying the QTLs. Those QTLs include two QTLs for seed weight on A5 (*TSWA5b* and *TSWA5c*), one for oleic acid content on A5 (*OLEA5*), and two for linolenic acid content on A4 (*LNAA4*) and C4 (*LNAC4*), respectively [11,47]. To test whether the comparative map could be used to target the candidate genes for mapped QTLs, we searched for the candidate genes underlying the mapped QTLs. It was found that the F block on LG A5 contained a *FAD2* gene for *OLEA5 ,* the H/J blocks on LG A4 and C4 harbored a *FAD3* gene for *LNAA4* and *LNAC4*, respectively (Figure 3A and 3C, column a). Similarly, the *MINI3* gene for *TSWA5b*, *TTG2* gene for *TSWA5c* could be predicted (Figure 3B, column a). Above predictions were exactly same as the previous analysis through homology cloning (Figure 3, column b) [11,47], demonstrating that the constructed comparative map can be effectively used in identification of candidate genes of mapped QTLs.

### Locating the putative homologous genes for seed weight/size on *B. napus* genetic map by comparative mapping

Sequence information was collected for 43 genes involved in the regulation of seed/fruit size or weight previously reported in tomato, maize, rice and *Arabidopsis* (Table 3). The ORF sequences of the genes were used for BLAST analysis against the *B. rapa* and *B. oleracea* genome. In total, 286 corresponding genes/loci in the two species were obtained with 132 from *B. rapa* and 154 from *B. oleracea*, respectively (Table 3; Additional file 9: Table S4). Among the 286 loci, 244 corresponded to the 35 seed size genes from *Arabidopsis* and the rest of 42 to the 8 genes from other species (Table 4; Additional file 9: Table S4).

**Table 3:** **Genes involved in seed size/weight regulation and their homologues in *B. rapa and B. oleracea***

**Gene**	**Species**	**Ref.**	**Copy number**		**Gene**	**Species**	**Ref.**	**Copy number**	
			***B. rapa***	***B. oleracea***				***B. rapa***	***B. oleracea***
*fw2.2*	Tomato	[48]	**6**	**4**	*AGL61*	*Arabidopsis*	[49]	**5**	**4**
*MN1*	Maize	[50]	**1**	**2**	*AGL62*	*Arabidopsis*	[51]	**8**	**7**
*CR4*	Maize	[52]	**1**	**1**	*AHP1*	*Arabidopsis*	[53]	**1**	**1**
*GW2*	Rice	[54]	**2**	**3**	*AHP2*	*Arabidopsis*	[53]	**4**	**8**
*GS3*	Rice	[55]	**0**	**2**	*AHP3*	*Arabidopsis*	[53]	**4**	**7**
*GW5*	Rice	[56,57]	**0**	**0**	*AHP4*	*Arabidopsis*	[53]	**3**	**2**
*GS5*	Rice	[18]	**5**	**5**	*AHP5*	*Arabidopsis*	[53]	**1**	**1**
*GIF1*	Rice	[58]	**3**	**7**	*AHP6*	*Arabidopsis*	[53]	**0**	**1**
*MINI3*	*Arabidopsis*	[59]	**4**	**7**	*ANT*	*Arabidopsis*	[60]	**6**	**5**
*AP2*	*Arabidopsis*	[61]	**3**	**2**	*CKI1*	*Arabidopsis*	[62]	**2**	**2**
*IKU1*	*Arabidopsis*	[63]	**3**	**3**	*DDM1*	*Arabidopsis*	[64]	**2**	**8**
*IKU2*	*Arabidopsis*	[59]	**1**	**3**	*EMS1*	*Arabidopsis*	[65]	**2**	**1**
*SHB1*	*Arabidopsis*	[66]	**4**	**6**	*FIS3*	*Arabidopsis*	[67]	**5**	**6**
*ARF2*	*Arabidopsis*	[68]	**8**	**4**	*FIS2*	*Arabidopsis*	[69]	**0**	**1**
*TTG2*	*Arabidopsis*	[70]	**7**	**8**	*GASA4*	*Arabidopsis*	[71]	**2**	**2**
*AHK1*	*Arabidopsis*	[72]	**1**	**1**	*FIS1*	*Arabidopsis*	[67]	**1**	**2**
*AHK2*	*Arabidopsis*	[72]	**2**	**2**	*MET1*	*Arabidopsis*	[73]	**3**	**3**
*AHK3*	*Arabidopsis*	[72]	**3**	**4**	*MSI1*	*Arabidopsis*	[67]	**6**	**7**
*AHK4*	*Arabidopsis*	[72]	**4**	**3**	*RGE1*	*Arabidopsis*	[74]	**2**	**2**
*AHK5*	*Arabidopsis*	[72]	**6**	**5**	*SHK1*	*Arabidopsis*	[75]	**4**	**5**
*KLU*	*Arabidopsis*	[9]	**3**	**3**	*DA1*	*Arabidopsis*	[76]	**2**	**2**
*AGL80*	*Arabidopsis*	[77]	**2**	**2**					
**Total**					**43**			**132**	**154**

**Table 4:** **Number of homologous genes for seed size/weight in *B. napus***

**LG**	**Gene origin**		**LG**	**Gene origin**	
	***Arabidopsis***	**Other species**		***Arabidopsis***	**Other species**
A1	8	2	C1	12	1
A2	8	2	C2	8	2
A3	14	1	C3	16	4
A4	8	2	C4	10	3
A5	15	2	C5	14	3
A6	14	4	C6 (BoC7) ^a^	7	3
A7	1	0	C7 (BoC6) ^b^	19	1
A8	9	0	C8	12	4
A9	14	4	C9	8	0
A10	10	0			
Scaffold	13	1	Scaffold	24	3
**Total**	**114**	**18**	**Total**	**130**	**24**

^a^ The homologous gene number was calculated based on the distribution on BoC7 because the LG C6 of *B. napus* has a high colinearity with BoC7.

^b^ The homologous gene number was calculated based on the distribution on BoC6 because the LG C7 of *B. napus* has a high colinearity with BoC6*.*

The copy numbers of the corresponding homologues in *B. rapa* and *B. oleracea* genomes varied. On average, one gene had 3 copies in the A genome and 3.6 copies in the C genome (Table 3). *B. rapa* and *B. oleracea* homologues for all genes were identified except for the gene *qSW5*/*GW5* from rice (Table 3).

Based on the colinear relationship between the A and C genome in three species (Table 1), the homologous genes were mapped onto the *B. napus* linkage map except the genes that currently are located only in the scaffolds of the *B. rapa* and *B. oleracea* genomes (Table 4; Figures 1 and 2, column d). Because of the fact that the LG C6 of *B. napus* was colinear with BoC7, and the LG C7 was colinear with BoC6, the homologous genes from BoC6 were placed on the LG C7 and BoC7 on LG C6, respectively (Figure 2). In total, 227 homologous genes of seed size/weight were finally positioned on the *B. napus* linkage map, which distributed on all LGs except A9 (Figures 1, 2; Table 4). Among the 185 *Arabidopsis* homologous genes mapped, 100 genes fell in conserved *Arabidopsis* genomic blocks and can be positioned exactly on the *B. rapa*/*B. oleracea* chromosomes (Figures 1 and 2, genes with asterisk). There were 20 homologous genes with tandem repeats (TR), among which three were from the homologous genes in crop species and 17 from *Arabidopsis*. Seventeen TRs genes were anchored onto the linkage map and the other three located in scaffolds of *B. rapa/B. oleracea* (Figures 1 and 2).

### Identification of the candidate genes underlying QTLs for seed weight in *B. napus*

The seed size/weight gene distribution map was used to identify candidate genes for the TSW QTLs by aligning the seed weight genes with the TSW QTL loci on the *B. napus* genetic map (Figures 1 and 2). On LG A1, *TTG2* and *GS5* were the nearest genes on each side of *TSWA1* (Figure 1 and Additional file 9: Table S4); *GW2* was located at the same position of *TSWA2* (Figure 1 and Additional file 9: Table S4); *CKI1* and *MN1* were nearby the peak of *TSWA4* (Figure 1 and Additional file 9: Table S4). *MINI3* and *FIE* were located nearby previously mapped *TSWA5a* and *TSWA5b*. The *MINI3* gene was located in the confidence interval of *TSWA5b,* consistent to our previous results [11], while the *FIE* gene fell into the confidence interval of *TSWA5a* (Figure 1 and Additional file 9: Table S4). Three genes, *AHP3*, *AHP5* and *MEA*, were located in the same confidence interval of *TSWA10* (Figure 1 and Additional file 9: Table S4). In addition, *AGL62*, *GS3* and *GASA4* were located on the peaks or in the confidence intervals of three newly identified QTLs, *TSWC2a*, *TSWC2b* and *TSWC2c*, respectively (Figure 2 and Additional file 9: Table S4). Together, above analysis clearly pinpointed the potential target genes for the mapped QTLs, providing valuable clues for a further comparison of sequence differences between two parental lines.

However, for the two major QTLs, *TSWA7a* and *TSWA7b*, no known information about candidate genes could be inferred from the map, suggesting that those QTLs may be unique in *B. napus* or *B. rapa* genome. The only one seed size/weight gene, *DDM1*, on LG A7 was far away from these two QTLs, thus unlikely the candidate gene for the two QTLs (Figure 1).

### Molecular cloning of *BnAP2* gene and development of gene-specific marker

**Figure 4:**
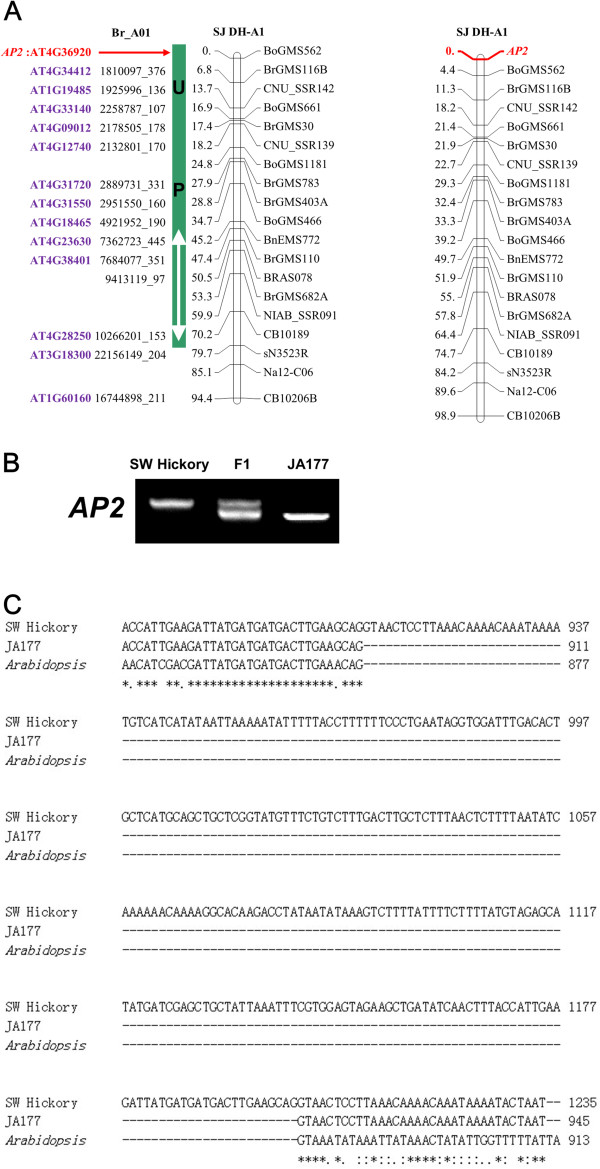
**Cloning of the *BnAP2 *gene with the aid of the comparative map.A**) Localization of the *BnAP2* gene and its allele-specific marker on LG A1. The red arrow on the left panel marks the *AP2* position as predicted in conserved block U. The right panel is a reconstructed LG A1 with the *BnAP2* allele-specific marker. **B**) PCR products amplified from the parental lines and their F_1_. The PCR products are separated by electrophoresis in 1.0% agarose gels and stained with ethidium bromide. **C**) Sequence difference of the *BnAP2* gene sequences between the two parental lines. There is a 290bp insertion in SW Hickory.

The *Arabidopsis AP2* (*AtAP2*, AT4G36920) gene has been reported to affect seed size [61]. *AtAP2* was located on the U-block of LG A1 in the comparative map (Figure 4A). Although no mapped QTL is matched to the predicted *AP2* gene, we reasoned that it is possible to find polymorphism of the locus between the two parental lines. To test this hypothesis, we set out to clone the homologous gene of *AtAP2* in *B. napus.* We searched for the homologous sequences in *B. rapa* genome with BLASTn by using AT4G36920 as a query. Primers were designed based on the retrieved sequence of *BrAP2* gene in *B. rapa* and the *BnAP2* gene was cloned in the two parental lines of the SJ DH population, respectively (Figure 4B and 4C; Additional file 10: Figure S5). Sequence analysis showed that the allele in SW Hickory contained a 290bp insertion compared to JA177 (Figure 4C and Additional file 10: Figure S5). A *BnAP2* gene-specific marker was developed based on the polymorphism between the two parents and used to map the gene in the SJ-DH population again. Genetic linkage analysis eventually mapped the *BnAP2* gene-specific marker onto the U block on LG A1, consistent with the predicted result on the comparative map (Figure 4A).

## Discussion

In this study, we developed a procedure for comparative mapping between *B. napus* and *Arabidopsis* with SSR markers with the aid of *B. rapa* and *B. oleracea* genome sequences. To the best of our knowledge, this is the first report to construct a comparative map among *Arabidopsis* and three *Brassica* species with a SSR-based genetic map (Additional files 3, 4, 5 and 6: Figure S1-S4; Additional file 1: Table S1). The SSR markers have been widely used as a preferable type of molecular marker in genetic mapping in *Brassica* species. However, it was difficult to use a SSR map for comparative mapping with *Arabidopsis* directly. First, individual SSR primer pairs only have limited sequence information, which renders a direct alignment with *Arabidopsis* genome ineffective. For example, in a study to construct a mainly SSR-based integrated map in *B. napus*, Wang *et al.*[32] found that <2% of the primer pairs could identify homologous regions to *Arabidopsis*, of which only 50% agreed with those identified using the corresponding SSR clone sequences. Second, high homology between the A and C genomes often results in multiple polymorphic loci in *B. napus* for a single *Arabidopsis* gene, which further complicates the comparative analysis between *B. napus* and *Arabidopsis*. In this study, we circumvented the two difficulties by making use of recently released genomic sequences of *B. rapa* and *B. oleracea.* Through anchoring the SSR loci on *B. napus* LGs to the *B. rapa/B. oleracea* genome by e-PCR, we were able to match the *B. napus* SSR loci with their *Arabidopsis* homologues, thus making such a comparative mapping feasible. By overcoming the difficulties in comparative mapping using a SSR-based genetic map of *B. napus* and *Arabidopsis* genomic sequences, this procedure thus proved a novel idea for a comprehensive comparison among *Arabidopsis*, *B. napus* and its two progenitor species, *B. rapa* and *B. oleracea*.

To make use of the information derived the SSR loci as much as possible, a less stringent E-value was initially used in this study to identify more putative homologous loci. As indicated by Lukens *et al.*[29], a less stringent cutoff could result in more non-specific region of homology. However, since our major purpose in this study was to establish colinear relationships between *B. napus* and *Arabidopsis* through the conserved blocks, such non-specific homology regions in the initial screening will be re-examined. With the criterion for identification of conserved blocks, such non-specific loci will not affect the determination of the conserved blocks. This is evident through the data listed in Additional file 1: Table S1, in which about 66% of the loci under the less stringent (E-value >1E-05) cutoff eventually were linked to a perspective block, indicating that some weak but biologically relevant sequence relationships could be revealed with such a procedure, which reduces the loss of valuable information from the SSR loci on the *B. napus* map.

The establishment of such a comparative map offers an effective way to transfer the gene information from model plant *Arabidopsis* to *B. napus*, an amphidiploid crop species, as demonstrated by mapping the seed size/weight genes on the *B. napus* genetic map (Figures 1 and 2). Furthermore, we identified candidate genes for eight TSW QTLs through the mapping (Figures 1 and 2; Additional files 3, 4, 5 and 6: Figure S1-S4). Together, the seed distribution map and the identified candidate genes for mapped TSW QTLs provide valuable information about the genetic control of seed weight in *B. napus.* Although such a list of seed size/weight genes could be further expanded by including other genes related to the process of seed development, our results do exemplify the universal usefulness of such an approach. A flow diagram for the process is presented in Additional file 11: Figure S6.

Mapping of the seed weight related genes and the candidate genes for TSW QTLs could accelerate the molecular cloning and functional characterization of the QTLs. As shown in Figure 3, the prediction of the candidate genes for several mapped QTLs is accurate. Such a process will allow us to isolate the potential candidate genes for a particular QTL by homologous cloning strategy rather than tedious and time-consuming traditional map-based cloning procedure. On the other hand, by cloning some of predicted potential candidate genes that were even not located in the genetic map, for example *AP2* in this study, it is possible to uncover the polymorphic alleles in two parental lines without QTL mapping information (Figure 4). By doing so, we were able to develop an allele-specific marker for one of locus of the *AP2* gene in *B. napus* and place the marker on the corresponding LG (Figure 4). There are three and two copies of the *AtAP2* homologues identified in *B. rapa* (including one copy located on a scaffold) and *B. oleracea*, respectively (Table 3; Additional file 9: Table S4). Consistently, there are four copies mapped on LGs A1, A3, C1 and C7 of the *B. napus* genetic map, respectively (Figures 1 and 2). Although the exact molecular significance of the insertion in the cloned *BnAP2* allele of SW Hickory is yet to be established, identification of the polymorphic locus between the two parental lines lays foundation for further functional characterization of all the *AP2* alleles in the *B. napus* genome.

The seed weight genetic map revealed the complexity of the genetic control of seed weight in amphidiploid rapeseed. For example, a single TSW QTL may have one or multiple candidate gene(s), such as *TSWA2* (with only one gene, *GW2,* located) and *TSWA1* (with 6 genes located) (Figure 1 and Additional file 9: Table S4). Mapping of these candidate genes could apparently narrow down the range of the potential target genes. Of course, even though potential candidate genes are mapped to a locus, this does not imply that they control the trait. The QTL may result from variation in other novel genes which have not been studied in model systems.

It is interesting to notice that some genes showing major effects on seed size/weight in rice and *Arabidopsis*, such as *GS3*, *GS5*, *GW2* and *MINI3*, *TTG2*, *ARF2*, *IKU2*, were located on the minor QTLs regions, or even not in the confidence intervals of previously mapped TSW QTLs (Figures 1 and 2; Additional file 9: Table S4). In addition, no homologue of *qSW5*/*GW5,* an important rice seed size gene [56,57] could be identified in both the A- and C- genome (Table 3). A more comprehensive evaluation of *B. napus* germplasm is needed to understand whether these genes may exhibit different effects on the studied trait in various species. On the other hand, no candidate genes for two previously mapped major QTLs, *TSWA7a* and *TSWA7b* were identified, suggesting that the two QTLs may represent novel determinants for seed weight in amphidiploid *B. napus*.

## Conclusions

We developed a procedure for comparative mapping between *B. napus* and *A. thaliana* with SSR markers with the aid of *B. rapa* and *B. oleracea* genome sequences. Such a SSR-based genetic map can be used to pinpoint the candidate genes for QTLs important in crop improvement. The procedure may also find wide applicability in Brassicaceae and other crop species, as candidate genes for QTLs in other pathways could be identified through a similar approach.

## Competing interests

The authors declare that they have no competing interests.

## Authors’ contributions

YZ and GC conceived the research. GC, YY, QY, ZZ, HC, JW and CF performed genetic mapping and collected the seed gene data. YY wrote the computer program and assisted in bioinformatic analysis. GC and YY performed the comparative mapping. GC carried out the molecular cloning. GC and YZ wrote the paper. All authors read and approved the final manuscript.

## Supplementary Material

Additional file 1 Table S1 Genetic linkage groups of *B. napus*, SSR marker primer sequences, amplification copy numbers, amplicon position in *B. rapa/B. oleracea* and homologous colinear locus in *Arabidopsis.* Click here for file

Additional file 2 The Perl script of e-PCRmap computer program. Click here for file

Additional file 3 Figure S1 Comparative map of *B. napus* with *A. thaliana* (LGs A1, A2, C1 and C2). Column a presents the genetic linkage groups (LGs) of the SJ DH population. LGs are represented by vertical bars with the loci position (in cM) indicated on the left and SSR loci names on the right. Column b and c list the homologous colinear loci in *B. rapa* and *B. oleracea*, respectively. The number means the physical position in *B. rapa* or *B. oleracea* chromosome with the length of amplification fragment. Column d and e are *Arabidopsis* gene codes corresponding to the homologous loci. Column f is the *Arabidopsis* conserved blocks identified in *B. napus*, which is nomenclatured according to Schranz *et al.*[5] and colored differently based on the *A. thaliana* (At) chromosome positions defined by Parkin *et al.*[30]. Inversions in the linkage groups relative to *Arabidopsis* are indicated by arrows. Column g lists the homologous genes of seed size or weight in *B. rapa* and *B. oleracea*. Genes with asterisk indicate that they are in good fit into both *Arabidopsis* and *B. rapa*/*B. oleracea* physical positions, and genes with brackets are the tandem repeats (TR) of homologous genes with the tandem repeat times in brackets. Click here for file

Additional file 4 Figure S2 Comparative map of *B. napus* with *A. thaliana* (LGs A3, A4 C3 and C4). Click here for file

Additional file 5 Figure S3 Comparative map of *B. napus* with *A. thaliana* (LGs A5-A7 and C5-C7). Click here for file

Additional file 6 Figure S4 Comparative map of *B. napus* with *A. thaliana* (LGs A8-A10, C8 and C9). (TIFF 1406 kb) Click here for file

Additional file 7 Table S2 QTLs for TSW in the SJ DH population detected previously and re-scanned in the present study. Click here for file

Additional file 8 Table S3 Distribution of conserved *Arabidopsis* genomic blocks in *B. napus* genome. Click here for file

Additional file 9 Table S4 Homologous genes of seed size/weight in *B. rapa* and *B. oleracea* chromosomes and scaffolds. Click here for file

Additional file 10 Figure S5 Comparative analysis of *AP2* genomic sequences of SW Hickory and JA177 with *Arabidopsis.* Click here for file

Additional file 11 Figure S6 A flow diagram for construction of a comparative map between *B. napus* with *Arabidopsis* based on the *B. rapa* and *B. oleracea* genome sequences, and for prediction of candidate genes for QTLs mapped on the map. Click here for file
