# Supplementary figures and images for: Identification of candidate genes of QTLs for seed weight in Brassica napus through comparative mapping among Arabidopsis and Brassica species
Source: BMC Genet. 2012 Dec 6;13:105. doi: 10.1186/1471-2156-13-105 (PMC3575274; doi:10.1186/1471-2156-13-105)

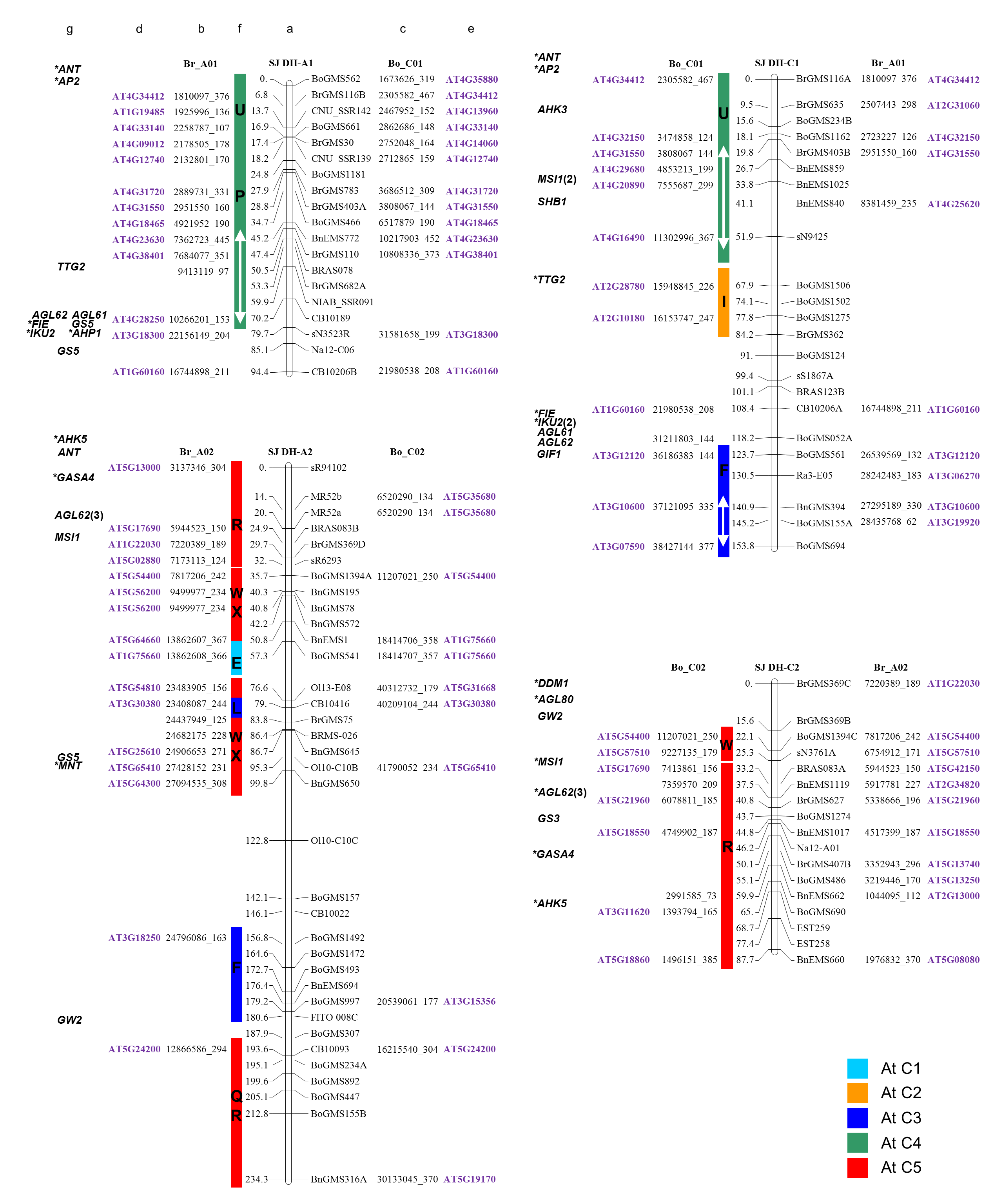

Supplement: Additional file 3 Figure S1 — Comparative map of B. napus with A. thaliana (LGs A1, A2, C1 and C2). Column a presents the genetic linkage groups (LGs) of the SJ DH population. LGs are represented by vertical bars with the loci position (in cM) indicated on the left and SSR loci names on the right. Column b and c list the homologous colinear loci in B. rapa and B. oleracea, respectively. The number means the physical position in B. rapa or B. oleracea chromosome with the length of amplification fragment. Column d and e are Arabidopsis gene codes corresponding to the homologous loci. Column f is the Arabidopsis conserved blocks identified in B. napus, which is nomenclatured according to Schranz et al.[5] and colored differently based on the A. thaliana (At) chromosome positions defined by Parkin et al.[30]. Inversions in the linkage groups relative to Arabidopsis are indicated by arrows. Column g lists the homologous genes of seed size or weight in B. rapa and B. oleracea. Genes with asterisk indicate that they are in good fit into both Arabidopsis and B. rapa/B. oleracea physical positions, and genes with brackets are the tandem repeats (TR) of homologous genes with the tandem repeat times in brackets. [file 1471-2156-13-105-S3.tiff]

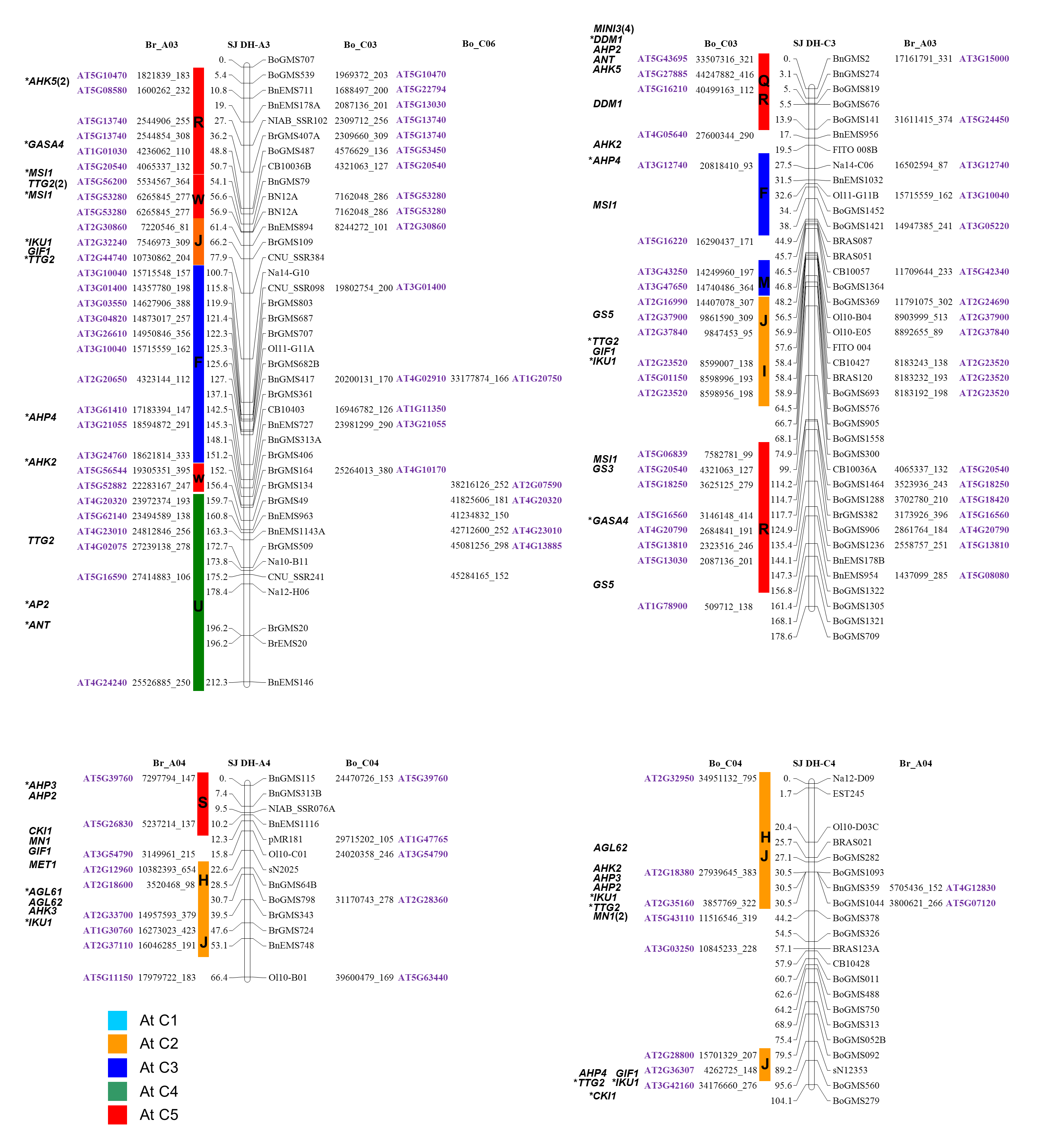

Supplement: Additional file 4 Figure S2 — Comparative map of B. napus with A. thaliana (LGs A3, A4 C3 and C4). [file 1471-2156-13-105-S4.tiff]

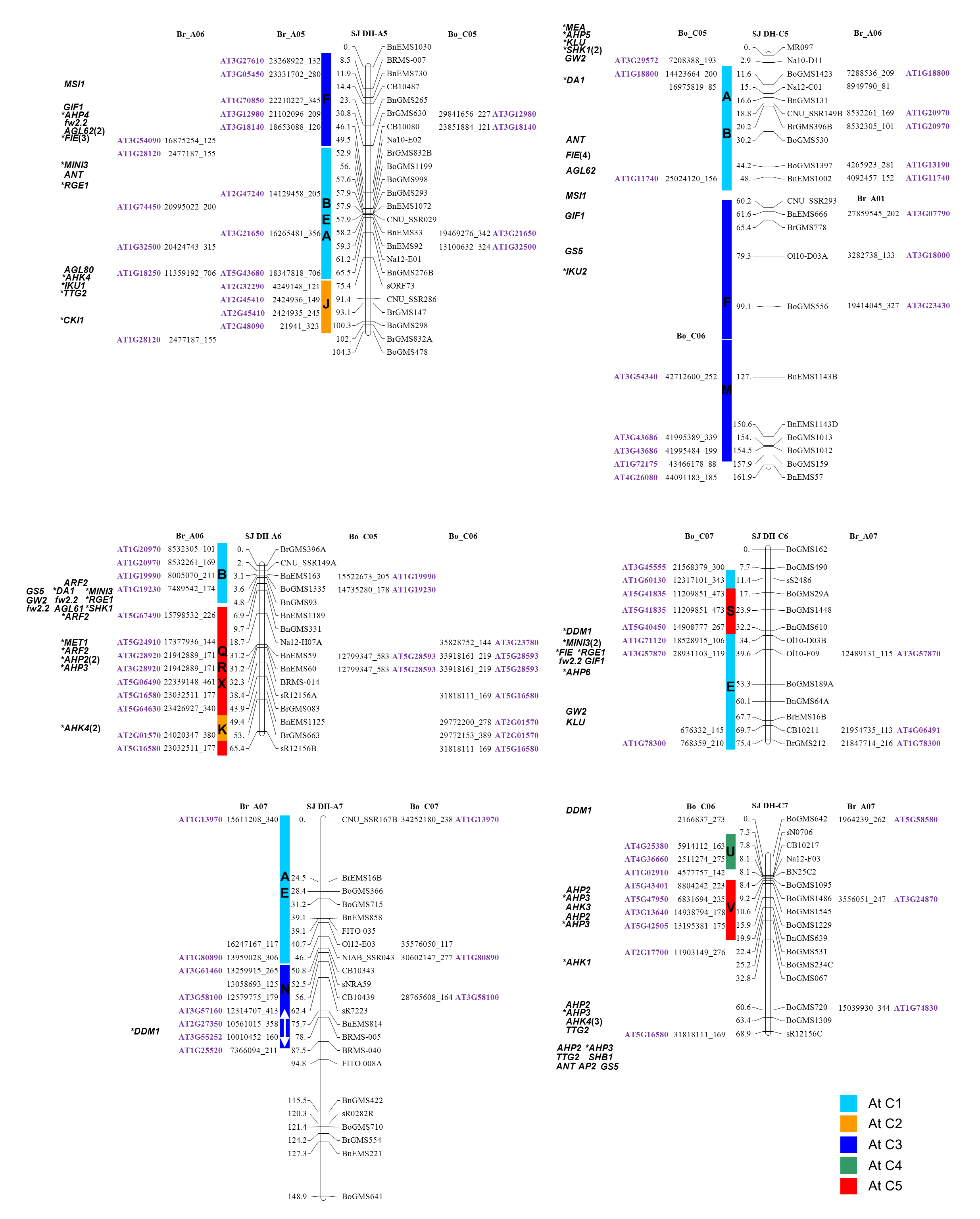

Supplement: Additional file 5 Figure S3 — Comparative map of B. napus with A. thaliana (LGs A5-A7 and C5-C7). [file 1471-2156-13-105-S5.tiff]

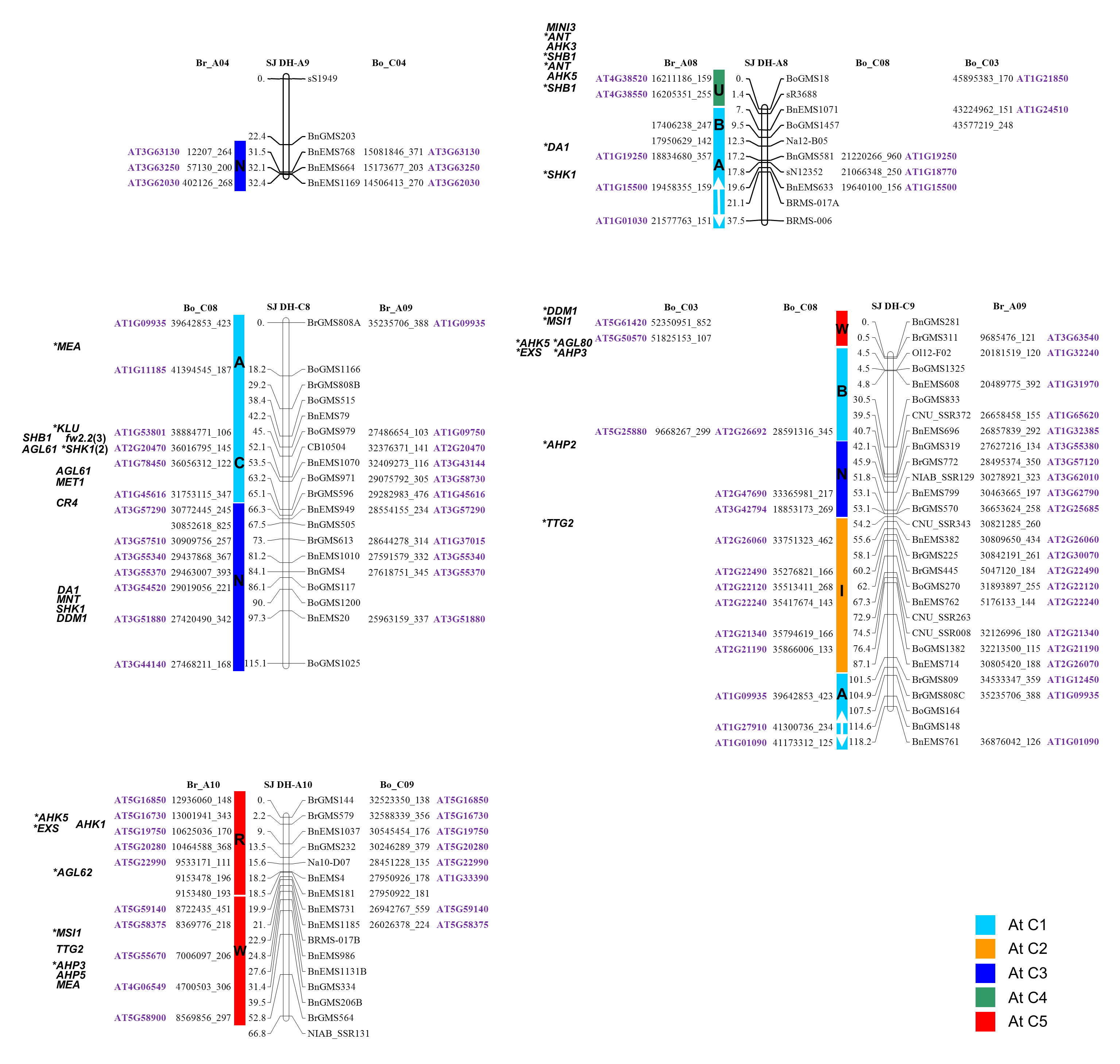

Supplement: Additional file 6 Figure S4 — Comparative map of B. napus with A. thaliana (LGs A8-A10, C8 and C9). (TIFF 1406 kb) [file 1471-2156-13-105-S6.tiff]

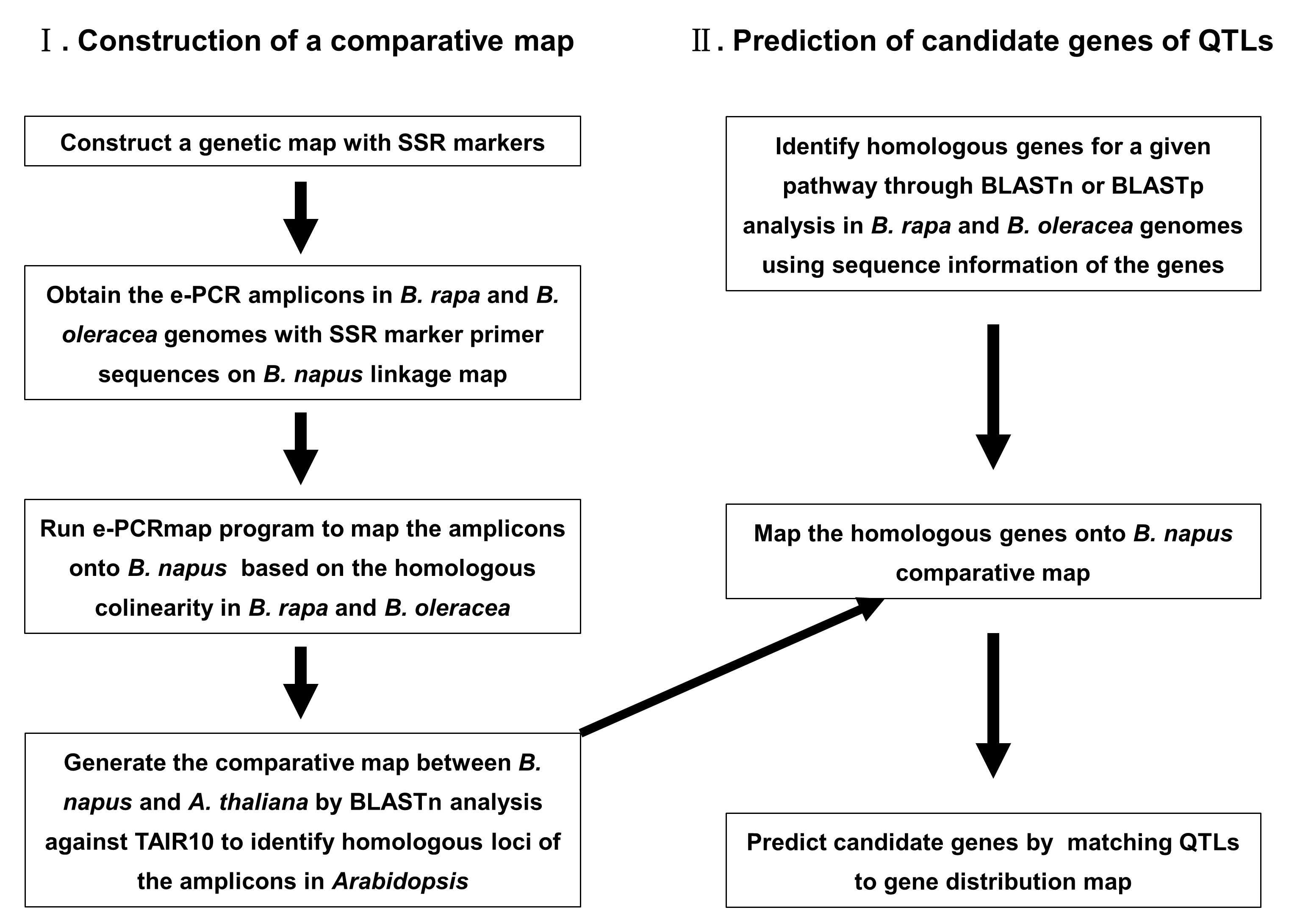

Supplement: Additional file 11 Figure S6 — A flow diagram for construction of a comparative map between B. napus with Arabidopsis based on the B. rapa and B. oleracea genome sequences, and for prediction of candidate genes for QTLs mapped on the map. [file 1471-2156-13-105-S11.tiff]
