# Supplementary material for: Identification of candidate genes of QTLs for seed weight in Brassica napus through comparative mapping among Arabidopsis and Brassica species
Source: BMC Genet. 2012 Dec 6;13:105. doi: 10.1186/1471-2156-13-105 (PMC3575274; doi:10.1186/1471-2156-13-105)
Supplement: Additional file 10 Figure S5 — Comparative analysis of AP2 genomic sequences of SW Hickory and JA177 with Arabidopsis. [file 1471-2156-13-105-S10.pdf]

```
SW Hickory      ATGTGGGATCTAAACGACTCACCACACCAAACACTTGTTGAAGAAGAATCTGAAGAGTTG 60
JA177           ATGTGGGATCTAAACGACTCACCACACCAAACACTTGTTGAAGAAGAATCTGAAGAGTTG 60
Arabidopsis     ATGTGGGATCTAAACGACGCACCACACCAAACACAAAGAGAAGAAGAATCTGAAGAGTTT 60
                ****************** ***************::.  :********************

SW Hickory      TGTTATTCTTCACCGGGTAAACGGGTCGGATCTTTCTCGAACTCAAGTTCATCTGCTGTA 120
JA177           TGTTATTCTTCACCGGGTAAACGGGTCGGATCTTTCTCGAACTCAAGTTCATCTGCTGTA 120
Arabidopsis     TGTTATTCTTCACCAAGTAAACGGGTTGGATCTTTCTCTAATTCAAGCTCTTCAGCTGTT 120
                **************..********** *********** ** ***** **:**:*****:

SW Hickory      GTCATCGAAGATGGATCCGATGATGATGAGCCTAACCGGGTCAGACCCAACAACCCACTT 180
JA177           GTCATCGAAGATGGATCCGATGATGATGAGCCTAACCGGGTCAGACCCAACAACCCACTT 180
Arabidopsis     GTTATCGAAGATGGATCCGATGACGATGAACTTAACCGGGTCAGACCCAATAACCCACTT 180
                ** ******************* *****.* ***************** *********

SW Hickory      GTCACCCATCAGTTCTTCCCACAGATGGAAACTAGCGTAGGGGATGATGGTGGAGGTGGT 240
JA177           GTCACCCATCAGTTCTTCCCACAGATGGAAACTAGCGTAGGGGATGATGGTGGAGGTGGT 240
Arabidopsis     GTCACCCATCAGTTCTTCCCTGAGATGGATTCTAACGGCGGTGGTGTTGCT--------- 231
                ********************: *******::***.** .** *.**:** *

SW Hickory      CCCGGGTCGGGCTTTCCTCGGTCTCACTGGTTTGGTGTTAAGTTTTGTCAGTCCGATCTA 300
JA177           CCCGGGTCGGGCTTTCCTCGGTCTCACTGGTTTGGTGTTAAGTTTTGTCAGTCCGATCTA 300
Arabidopsis     ------TCTGGCTTTCCTCGGGCTCACTGGTTTGGTGTTAAGTTTTGTCAGTCGGATCTA 285
                      ** ************ ******************************* ******

SW Hickory      GCTACCGGATCATCAGCGGGTAAACCCGCCACTGTAGCC---CCCGTGGCGGAGCCGGCT 357
JA177           GCTACCGGATCATCAGCGGGTAAACCCGCCACTGTAGCC---CCCGTGGCGGAGCCGGCT 357
Arabidopsis     GCCACCGGATCGTCCGCGGGTAAAGCTACCAACGTTGCCGCTGCCGTAGTGGAGCCGGCA 345
                ** ********.**.********* * .***.  **:***    ****.* *********:

SW Hickory      CAGCCGTTGAAAAAGAGCAGGCGTGGACCACGGTCGAGGAGTTCTCAGTATAGAGGCGTT 417
JA177           CAGCCGTTGAAAAAGAGCAGGCGTGGACCACGGTCGAGGAGTTCTCAGTATAGAGGCGTT 417
Arabidopsis     CAGCCGTTGAAAAAGAGTCGGCGTGGACCAAGATCAAGAAGTTCTCAGTATAGAGGTGTT 405
                ***************** .***********.*.**.**.**************** ***

SW Hickory      ACGTTTTACCGGCGAACCGGAAGATGGGAATCTCATATTTGGTAATTTATTTGCAGTGAA 477
JA177           ACGTTTTACCGGCGAACCGGAAGATGGGAATCTCATATTTGGTAATTTATTTGCAGTGAA 477
Arabidopsis     ACGTTTTACCGGCGTACCGGAAGATGGGAATCTCATATTTGGTAAT-------------A 452
                **************:*******************************             *

SW Hickory      AAAATATTCTTTAAATTTGATTAATCGATCGTTAATTAAAAGATAATGAATG-------T 530
JA177           AAAATATTCTTTAAATTTGATTAATCGATCGTTAATTAAAAGATAATGAATG-------T 530
```

```
Arabidopsis     ATCTCATATTTTTAATTTCGTTAATCGATCGTACTTTAGATTATAAATTTAAGTTTTTTT 512
                *:.: **: ***:***** .************:.:***.*: ****: :::.       *

SW Hickory      AAGATATGTTTCTCTATAATTCAGGGACTGTGGGAAGCAGGTTTACTTAGGTATATTAAC 590
JA177           AAGATATGTTTCTCTATAATTCAGGGACTGTGGGAAGCAGGTTTACTTAGGTATATTAAC 590
Arabidopsis     TTTGTTTGTTCTTCTGAATTTCAGGGACTGTGGGAAACAAGTTTACTTAGGTAATT---- 568
                :: .*:****  ***.:*:*****************.**.*************::*

SW Hickory      TTTTTTTTGTTTTAG-TTTTTTTTTTCAT-TTTTTTCTTGTATTCTGCTGTTGAAAAATG 648
JA177           TTTTTTTTGTTTTAGTTTTTTTTTTTCAT-TTTTTTCTTGTATTCTGCTGTTGAAAAATG 649
Arabidopsis     ----------------TTATTTTCCTCATGTTTTTTTTTGTATTTTGGTGTTGAAAAATG 612
                                **:****  **** ****** ******* ** ************

SW Hickory      TCATCATATAATTAAAAATATTTTTACCTTTTTTCCCTGAATAGGTGGATTTGACACTGC 708
JA177           TCATCATATAATTAAAAATATTTTTACCTTTTTTCCCTGAATAGGTGGATTTGACACTGC 709
Arabidopsis     TCATCATA-ATTTTAATTTATTATAATCT-------CTGAATAGGTGGATTTGACACTGC 664
                ******** *:**:**::****:*:* **       ***********************

SW Hickory      TCATGCAGCTGCTCGGTATGTTTCTGTCTTTGACTTGCTCTTTAAC-------TCTTTTA 761
JA177           TCATGCAGCTGCTCGGTATGTTTCTGTCTTTGACTTGCTCTTTAAC-------TCTTTTA 762
Arabidopsis     TCATGCAGCAGCTCGGTATTTTTCTCTCTTTGACTCTCTCTATATTGAGTTGTTATTTAT 724
                *********:********* ***** *********  ****:**:        *.***::

SW Hickory      ATATCAAAAAACAAAAGGCACAAGACCTATAATATAAAGTCTTTTATTTTC----TTTTA 817
JA177           ATATCAAAAAACAAAAGGCACAAGACCTATAATATAAAGTCTTTTATTTTC----TTTTA 818
Arabidopsis     TTATTTTTTAAAAAATACCGGAAGAAATTTATAAAAATTAATTTTAATTTTGTTTTATTT 784
                :*** ::::**.***:.  *.  ****..*:**::*:**: :.*****:***     *:**:

SW Hickory      TGTAGAGCATATGATCGAGCTGCTATTAAATTTCGTGGAGTAGAAGCTGATATCAACTTT 877
JA177           TGTAGAGCATATGATCGAGCTGCTATTAAATTTCGTGGAGTAGAAGCTGATATCAACTTT 878
Arabidopsis     AATAGAGCATATGATAGAGCTGCTATTAAATTCCGTGGAGTAGAAGCGGATATCAATTTC 844
                :.*************.**************** ************** ******** **

SW Hickory      ACCATTGAAGATTATGATGATGACTTGAAGCAGGTAACTCCTTAAACAAAACAAATAAAA 937
JA177           ACCATTGAAGATTATGATGATGACTTGAAGCAG--------------------------- 911
Arabidopsis     AACATCGACGATTATGATGATGACTTGAAACAG--------------------------- 877
                *.*** **.*******************.***

SW Hickory      TGTCATCATATAATTAAAAATATTTTTACCTTTTTTCCCTGAATAGGTGGATTTGACACT 997
JA177           ------------------------------------------------------------
Arabidopsis     ------------------------------------------------------------
```

```
SW Hickory      GCTCATGCAGCTGCTCGGTATGTTTCTGTCTTTGACTTGCTCTTTAACTCTTTTAATATC 1057
JA177           ------------------------------------------------------------
Arabidopsis     ------------------------------------------------------------

SW Hickory      AAAAAACAAAAGGCACAAGACCTATAATATAAAGTCTTTTATTTTCTTTTATGTAGAGCA 1117
JA177           ------------------------------------------------------------
Arabidopsis     ------------------------------------------------------------

SW Hickory      TATGATCGAGCTGCTATTAAATTTCGTGGAGTAGAAGCTGATATCAACTTTACCATTGAA 1177
JA177           ------------------------------------------------------------
Arabidopsis     ------------------------------------------------------------

SW Hickory      GATTATGATGATGACTTGAAGCAGGTAACTCCTTAAACAAAACAAATAAAATACTAAT-- 1235
JA177           ------------------------GTAACTCCTTAAACAAAACAAATAAAATACTAAT-- 945
Arabidopsis     ------------------------GTAAATATAAATTATAAACTATATTGGTTTTTATTA 913
                                        ****.*. ::*::.:****:*::::..*: *:**

SW Hickory      -CAATATGAATAGGTTATT---ATATTGATTAAAACGTAATTTAGCAGATGACGAATTTA 1291
JA177           -CAATATGAATAGGTTATT---ATATTGATTAAAACGTAATTTAGCAGATGACGAATTTA 1001
Arabidopsis     ACGATTTTTAAAGGTTTGGGAGATTAATATTGAAATTGAATTTTATAGATGACTAATTTA 973
                 *.**:* :*:*****:     **::: ***.***   *****:. ******* ******

SW Hickory      ACGAAGGAAGAGTTCGTGCACGTACTTCGCCGACAAAGCACAGGCTTCCCTCGAGGCAGT 1351
JA177           ACGAAGGAAGAGTTCGTGCACGTACTTCGCCGACAAAGCACAGGCTTCCCTCGAGGCAGT 1061
Arabidopsis     ACCAAGGAAGAGTTCGTACACGTACTTCGCCGACAAAGCACAGGCTTCCCTCGAGGAAGT 1033
                ** **************.**************************************.***

SW Hickory      TCAAAGTATAGAGGTGTCACTTTGCATAAGTGTGGTCGTTGGGAAGCTCGAATGGGTCAA 1411
JA177           TCAAAGTATAGAGGTGTCACTTTGCATAAGTGTGGTCGTTGGGAAGCTCGAATGGGTCAA 1121
Arabidopsis     TCGAAGTATAGAGGTGTCACTTTGCATAAGTGTGGTCGTTGGGAAGCTCGAATGGGTCAA 1093
                **.*********************************************************

SW Hickory      TTCTTAGGCAAAAAG--TACTTTCATTCATT-------CATTCTTTATTTTTCTAATAGA 1462
JA177           TTCTTAGGCAAAAAG--TACTTTCATTCATT-------CATTCTTTATTTTTCTAATAGA 1172
Arabidopsis     TTCTTAGGCAAAAAGTATAATTTCTCATTTTATATTCACTCGAAAACTTCATTTTTAG 1153
                ***************  **.****: *****       ** ** ::*: ** .*::*:..

SW Hickory      TTTGTTATTGTAACTTGAG--TTTTGATGCGTTAACTTTTCATATTAGGTATGTTTATTT 1520
```

```
JA177          TTTGTTATTGTAACTTGAG--TTTTGATGCGTTAACTTTTCATATTAGGTATGTTTATTT 1230
Arabidopsis    TTTGTTATTTTAACTTTGAGTTTTTGTTTCTTGAATCTTATAAAATAGGTATGTTTATTT 1213
               ********* ****** ..  *****:* * * **  **: *:*:***************

SW Hickory     GGGTTTGTTCGACACCGAGGTTGAAGCTGCTAGGTAAATGTCTTTCTGATTGATTCCACA 1580
JA177          GGGTTTGTTCGACACCGAGGTTGAAGCTGCTAGGTAAATGTCTTTCTGATTGATTCCACA 1290
Arabidopsis    GGGTTTGTTCGACACCGAGGTCGAAGCTGCTAGGTAAATGTCTTTTTGTTTGATTCTACA 1273
               ********************* *********************** **:******* ***

SW Hickory     ACACACATTGTT--AGAAAGCTTTAATCTCGT---ATAATGTTTTATTTGTTTTTTGA 1635
JA177          ACACACATTGTT--AGAAAGCTTTAATCTCGT---ATAATGTTTTATTTGTTTTTTGA 1345
Arabidopsis    ACACACATTGTTGTATAATGTGTTTTTCTCGTTACTAATTGATTTTCATTATTTTATATA 1333
               ************  * **:*  **::******    ::*:**:****.:**: ***:*: *

SW Hickory     TGACCACAGAGCTTACGATAAAGCTGCAATCAAATGTAATGGCAAAGACGCTGTGACTAA 1695
JA177          TGACCACAGAGCTTACGATAAAGCTGCAATCAAATGTAATGGCAAAGACGCTGTGACTAA 1405
Arabidopsis    TAATCACAGAGCTTACGATAAAGCTGCAATCAAATGTAACGGCAAAGACGCCGTGACCAA 1393
               *.* ********************************* *********** ***** **

SW Hickory     CTTTGATCCAAGCATATACGACGACGAATTGAATGCCGGTAATTTTTATTTCAAAATC-- 1753
JA177          CTTTGATCCAAGCATATACGACGACGAATTGAATGCCGGTAATTTTTATTTCAAAATC-- 1463
Arabidopsis    CTTTGATCCGAGTATTTACGATGAGGAACTCAATGCCGGTAAATTGTCTCATTTAATCGA 1453
               *********.** **:***** ** *** * ***********:** *.* :  ::****

SW Hickory     ------------TTTTTTGAGTTTTTAATCGTATCTTTTTGG-GAAAGAACTTGGTTTCA 1800
JA177          ------------TTTTTTGAGTTTTTAATCGTATCTTTTTGG-GAAAGAACTTGGTTTCA 1510
Arabidopsis    GTAATTTTATATATTTTTTTGGTCCTTAGTTTCATCTCTTAGGTGTTCGAACTTGGTTTAA 1513
                           :***** .**  ***.*   **** **:** *::.***********.*

SW Hickory     AGATTTTCTTTTTAAATTTGTGTAGAGTCATCAGGGAATCCTAT---TCAACAAGATCAT 1857
JA177          AGATTTTCTTTTTAAATTTGTGTAGAGTCATCAGGGAATCCTAT---TCAACAAGATCAT 1567
Arabidopsis    AGATTTTGAATTTGG-TGTATATAGAGTCATCAGGGAATCCTACTACTCCACAAGATCAC 1572
               ******* ::***..  * *.*.*********************    **.*********

SW Hickory     AACCTCGATTTGAGTTTGGGAAACTCGGTTAATTCGAAGCAAAAGGGTCAAGATATGCGG 1917
JA177          AACCTCGATTTGAGTTTGGGAAACTCGGTTAATTCGAAGCAAAAGGGTCAAGATATGCGG 1627
Arabidopsis    AACCTCGATTTGAGCTTGGGAAATTCGGCTAATTCGAAGCATAAAAGTCAAGATATGCGG 1632
               ************** ******** **** ************:**..**************

SW Hickory     CTCAAGGTAAAGTG-TTTATTAAATTATATACTATAGTTTCTACCTTAAATTT---CTTC 1973
JA177          CTCAAGGTAAAGTG-TTTATTAAATTATATACTATAGTTTCTACCTTAAATTT---CTTC 1683
Arabidopsis    CTCAGGGTAGAGTTTAATCTTATATTATTAACAATAATTTATATCTTAATATATAGTTTA 1692
```

```
                 ****.****.***  ::*.***:*****::**:***.***.** *****::*:    **.

SW Hickory       GTTTGTTATAAACATTTTTTTCTTTTTATT------AGATGAACCAACAA------GATT 2021
JA177            GTTTGTTATAAACATTTTTTTCTTTTTATT------AGATGAACCAACAA------GATT 1731
Arabidopsis      TATTGTTATAAACATGTTTTCTTTTGTTTTGCTTTCAGATGAACCAACAACAACAAGATT 1752
                  :************* ****  *** *:**      **************      ****

SW Hickory       CTCTTCATCCTAATGAGATTCTTGGATTGGGTCAAACCGGAATGGTTAACCATATCCCAA 2081
JA177            CTCTTCATCCTAATGAGATTCTTGGATTGGGTCAAACCGGAATGGTTAACCATATCCCAA 1791
Arabidopsis      CTCTCCACTCTAATGAAGTTCTTGGATTAGGTCAAACCGGAATGCTTAACCATACTCCCA 1812
                 **** **  *******..**********.*************** *********  **.*

SW Hickory       ATTCAAATCTCCAAGTGAGTAAAAAACACACACAAGAAGATATAAAACATTTGTTTAAAT 2141
JA177            ATTCAAATCTCCAAGTGAGTAAAAAACACACACAAGAAGATATAAAACATTTGTTTAAAT 1851
Arabidopsis      ATTCAAACCACCAAGTGAGTAAATAAC-CACAAATGCAAATACCAT-----------AAT 1860
                 ******* *:*************:*** ****.*:*.*.*** .*:           ***

SW Hickory       TTCTATCGGTTATGTTTTTACTAAAGAATATGTATATTTTTGTATGGTGTAAATTAGTTT 2201
JA177            TTCTATCGGTTATGTTTTTACTAAAGAATATGTATATTTTTGTATGGTGTAAATTAGTTT 1911
Arabidopsis      TTCATTTGAATATATTTTATCTAAAGAATTG---CATTTTTTTTTG--GTAAATTAGTTT 1915
                 ***::* *.:***.****::*********:     ****** *:**  ************

SW Hickory       CGGGGCAGCAGCAACATTGGTGGCGGAGGAGGATTCTCACTATTTCCGGTGGCTGAGAAC 2261
JA177            CGGGGCAGCAGCAACATTGGTGGCGGAGGAGGATTCTCACTATTTCCGGTGGCTGAGAAC 1971
Arabidopsis      CCGGGCAGCAGCAACATTGGTAGCGGAGGCGGATTCTCACTGTTTCCGGCGGCTGAGAAC 1975
                 * *******************.*******.***********.******* **********

SW Hickory       CACCGGTTTGATGGTCGGACCACGACGAACCAAGTGTTGGCAAATGCTGCAGCATCATCA 2321
JA177            CACCGGTTTGATGGTCGGACCACGACGAACCAAGTGTTGGCAAATGCTGCAGCATCATCA 2031
Arabidopsis      CACCGGTTTGATGGTCGGCCTCGACGAACCAAGTGTTGACAAATGCTGCAGCATCATCA 2035
                 ******************.**:*****************.********************

SW Hickory       GGATTCTCTCCTCATCATCACAATCAGATTTTTAATTCCACTTCTACTTCTCATCAAAAT 2381
JA177            GGATTCTCTCCTCATCATCACAATCAGATTTTTAATTCCACTTCTACTTCTCATCAAAAT 2091
Arabidopsis      GGATTCTCTCCTCATCATCACAATCAGATTTTTAATTCTACTTCTACTCCTCATCAAAAT 2095
                 ************************************** ********* ***********

SW Hickory       TGGCTGCAGACCAATGGCTTCCAACCTTCTCTCATGAGACCTTCTTGA 2429
JA177            TGGCTGCAGACCAATGGCTTCCAACCTTCTCTCATGAGACCTTC-TGA 2138
Arabidopsis      TGGCTGCAGACAAATGGCTTCCAACCTCCTCTCATGAGACCTTCTTGA 2143
                 ***********.*************** ****************.***
```

Additional File 10: Figure S5 Comparative analysis of *AP2* genomic sequences of SW Hickory and JA177 with *Arabidopsis*. *Underlined* was the sequence of *AP2* genomic primer.
